# An assessment of Individual, community and state-level factors associated with inadequate iodised salt consumption among pregnant and lactating women in Nigeria

**DOI:** 10.1186/s12884-023-05833-w

**Published:** 2023-07-18

**Authors:** Yusuf Olushola Kareem, Edward Kwabena Ameyaw, Roberta Mensima Amoah, Oyelola A Adegboye, Sanni Yaya

**Affiliations:** 1 United Nations Population Fund, Country Office, Abuja, Nigeria; 2 Institute of Policy Studies and School of Graduate Studies, Lingnan University, Lingnan, Hong Kong; 3 Maternal and Child Health Unit, Directorate of University Health Services, University for Development Studies, Tamale, Ghana; 4 Menzies School of Public Health, Charles Darwin University, Darwin, NT 0811 Australia; 5 School of International Development and Global Studies, University of Ottawa, Ottawa, Canada; 6 The George Institute for Global Health, Imperial College London, London, United Kingdom

**Keywords:** Salt, Iodine-deficiency, pregnant women, breastfeeding mothers, Cross-sectional survey, Multi-level analysis, Nigeria

## Abstract

**Background:**

Iodine deficiency is the most common cause of thyroid disease, and in its severe form can result in cretinism; the impairment of the brain development of a child. Pregnant and breastfeeding women’s daily iodine requirement is elevated due to physiological changes in iodine metabolism, requiring up to double the iodine intake of other women. Although Nigeria was the first African country to be declared iodine sufficient in 2007, recent evidence has shown that only about seven in ten households consume salt with adequate iodine content (≥ 15 ppm), with variation across states. The study aimed to assess the Individual- and household-, community- and state-level factors associated with inadequate iodised salt consumption among pregnant women and breastfeeding mothers in Nigeria.

**Methods:**

This study utilised the Multiple Indicator Cluster Survey to assess factors associated with inadequate iodised salt consumption among 4911 pregnant women and breastfeeding mothers in Nigeria. The descriptive analysis was presented using frequencies and percentages. The prevalence of adequate and inadequate iodised salt consumption with their 95% confidence interval were computed. Several multi-level mixed effect log-binomial logistic regressions were used to explore the factors associated with inadequate iodised salt consumption. The Loglikelihood, Akaike Information Criterion and Bayesian Information Criterion were used to assess the goodness of fit of the models. All analyses were adjusted for the complex survey design and analysed using Stata 15.0 at p < 0.05.

**Results:**

The prevalence of inadequate iodised salt consumption among pregnant and breastfeeding mothers was 35.2% (95% CI: 33.1–37.5). Inadequate consumption of iodised salt was highest among pregnant and breastfeeding women aged 45–49 years (48.2%; 95%CI: 37.8–58.8), as well as those with non-formal education (52.7%; 95%CI: 47.7–57.6) and no education (34.6%; 95%CI: 31.3–38.1). Our findings revealed that pregnant and breastfeeding women living in the poorer, middle, richer and richest quintiles were 32%, 47%, 35% and 62% less likely to consume salt with inadequate iodine compared to those in the poorest households. Those with non-formal education were 1.8 times (95%CI: 1.36–2.42) more likely to consume salt with deficient iodine than those without education. Pregnant and breastfeeding mothers residing in moderately and most deprived communities were 3.5 (95%CI: 2.57–4.73) and 4.7 times (95%CI: 3.38–6.55) more likely to consume salt with inadequate iodine than those from least deprived communities. Women in the Northwestern region and those from the Southwestern region were 4.0 and 3.5 times, respectively, more likely to consume salt with inadequate iodine compared to pregnant and breastfeeding women residing in the North-Central region.

**Conclusions:**

The study has shown that inadequate consumption of iodised salt dominates among older pregnant and breastfeeding women. Also, women with non-formal education have higher prospects of consuming salt with lesser iodine. There is a need to enhance women’s economic opportunities and empowerment as well as sensitisation on their nutritional requirements during pregnancy and breastfeeding. Both formal and non-formal educational initiatives on nutrition are extremely important and should be prioritised by the Nigerian government in its efforts to encourage the consumption of iodised salt among pregnant and lactating mothers. Additionally, health promotion interventions that seek to advocate iodised salt intake must be prioritised by the actors in the health sector.

**Supplementary Information:**

The online version contains supplementary material available at 10.1186/s12884-023-05833-w.

## Background

Iodine is an essential element that directly affects thyroid gland secretions, which is necessary for normal fetal growth and development, and to a great extent, control heart action, nerve response to stimuli, and improves the motor and cognitive functions of a child [1, 2]. Worldwide, iodine deficiency is the most common cause of thyroid disease. In its severe form, particularly during gestation and in the first months following the birth can result in cretinism, the impairment of the brain development of a child [3]. Fortunately, iodine deficiency can be prevented by adequate dietary intake of iodine, which is most often achieved by adding iodine to salt [3].

Salt is a compound composed primarily of sodium (Na) and chloride (Cl) and is of great importance to human and animal health [4, 5]. Sodium is an essential nutrient for human health via its role as an electrolyte and osmotic solute [5]. Iodised salt containing potassium iodide, is the most common source of natural forms of iodine, an essential micronutrient for normal human growth and development [6, 7]. World Health Organization (WHO) and the International Council for the Control of Iodine Deficiency Disorders (ICCIDD) had proposed salt iodisation strategy has a universal intervention to control and eliminate Iodine deficiency and advocated that adequately iodised salt must not only reach the entire affected population, but also those groups that are the most susceptible which are pregnant women, breastfeeding mothers and young children [3].

The landscape of Nigeria predisposes the country to iodine deficiency disorder considering the country’s proximity to the equator coupled with the long months of rainfall, typically from April to November [8]. Consequently, a number of interventions have been implemented to ensure that the populace achieve the recommended iodine intake. For instance, in 1992, the Standard Organization of Nigeria (SON) enacted that all food-grade salt should be iodised with 50 ppm potassium iodine when packaging. This was subsequently revised with an inclusion of 30 ppm at the distribution and retailing stages, as well as above 15 ppm iodine at household level [9, 10]. Besides, the National Agency for Food and Drug Administration and Control Act also indicates the required ingredients for iodised salt [11]. A previous study has reported an average of 43.71 parts per million (ppm) iodine in retail common salts, ranging from 38.45 to 49.57 ppm [12].

Pregnant women’s daily iodine requirement is elevated due to physiological changes in iodine metabolism [13, 14]. The recommended daily iodine intake is 150 µg/L for adults, 220 µg/L for pregnant women, and 290 µg/L for lactating mothers [3]. Adequate dietary intake of Iodine is critical for brain development, and iodine deficiency is the single most important preventable cause of brain damage and irreversible mental retardation [3, 7, 15, 16].

Despite the introduction of salt iodisation in many countries to control iodine deficiency, adverse effects of inadequate intake continue to be a problem, with an estimated 1.9 billion people at risk worldwide [17]. In Turkey, a study revealed that iodine deficiency still remains a serious problem among pregnant women after eight years of compulsory salt iodination in the country [18]. The majority of people using non-iodised salt in most Sub-Saharan African (SSA) countries are among poor, younger women and those who were pregnant [19]; but there exist geographical variations among countries. For example, the proportion of people with no iodised salt ranges from 29.5% in Senegal, 21.3% in Tanzania, 14% in Ethiopia, 11.6% in Malawi and 10.8% in Angola [19]. In addition, an assessment of iodine status among pregnant women in a rural community in Ghana revealed a prevalence of 42.5% iodine deficiency [20].

Although Nigeria was the first African country to be declared iodine sufficient in 2007, a recent national survey has shown that only about seven in ten households consume salt with adequate iodine content (≥ 15 ppm) with variation across states [21]. This implies that a greater proportion of household members, including pregnant women, do not consume salt with adequate iodine content. Additionally, some evidence has shown that most pregnant women tend to have inadequate iodine intake (60.5%) relative to nonpregnant women (27.3%) [22]. Besides, this varies along the pregnancy trajectory, as the authors noted that 53.6%, 59% and 72% pregnant women in the first, second and third trimesters had inadequate iodine intake, respectively [22]. In the case of breastfeeding women in Nigeria, there appears to be no evidence regarding their Iodine intake.

Some studies conducted at community and state levels have shown striking differences in iodine deficiency among women. For example, a study in Zaria, Northwestern Nigeria, revealed iodine sufficiency among pregnant women [23], while another study showed that residents of Nanka and Oba towns of Anambra State, Southeastern Nigeria, were at risk of iodine deficiency disorders [24]. Therefore, this study utilised national representative data to assess Individual- and household, community- and state-level factors associated with inadequate iodised salt consumption among pregnant women and breastfeeding mothers in Nigeria.

## Materials and methods

### Data sources

The Nigeria Multiple Indicator Cluster Survey (MICS) conducted between September 2016 to January 2017 was utilised for this study. The 2016-17 Nigeria MICS was designed to provide national, regional and state-level estimates and considered urban and rural differences. This cross-sectional survey is aimed at developing evidence-based policies and programmes and for monitoring progress toward national goals and global commitments. A two-stage sampling procedure was adopted using the National Integrated Survey of Households round 2 (NISH2) extracted from the 2006 Population Census as a sampling frame and the basis for selecting Enumeration Areas (EAs). The Primary Sampling Units (PSUs) consisted of EAs selected in each state, while households within each EAs were selected at the second stage. The states within each of the six geo-political regions were used as the sampling strata. The MICS 2016-17 used four types of questionnaires, namely household, women, men, and under-five children questionnaires to elicit information on demographic, household, women, men and children’s health indicators. Information on the availability of salt for cooking and also testing for its iodine content was included in the household questionnaire. Further details on sampling technique, data collection and administration are provided in the report [21].

### Outcome variable

In this study, we extracted information on the iodine content in salt among women who were pregnant or currently breastfeeding. The study population included 4,911 pregnant and breastfeeding mothers at the time of survey (72% of them are currently pregnant while 28% are breastfeeding mothers) in 3,976 households within 1,613 communities across 36 States including the Federal Capital Territory. The amount of iodine in salt samples were categorised into three, namely: [1] salt in the household does not contain iodine [2] more than 0 parts per million (ppm) and less than 15 ppm [3] 15 ppm or more. To investigate factors associated with salt consumption with inadequate iodine content; respondents who consumed salt containing 15 ppm iodine or more were considered as having adequate iodine intake, while those who consumed salt with no iodine or less than 15 ppm were deemed deficient in iodine. For this current MICS and other previous rounds of survey across other countries where salt iodisation test were conducted, the interviewers were trained on salt testing using a salt iodization test kit. The salt testing kits used in the MICS surveys distinguish only between less than 15 ppm versus 15 ppm or greater, in addition to 0 ppm. This is in conformity with the internationally agreed indicator for iodised salt, that categorized salt containing 15 parts per million (ppm) or more of iodate/iodide considered as being adequately iodized.

### Explanatory variables

The explanatory variables were grouped into Individual- and household-, community, and state-level factors. Individual factors considered were respondent age group (15–19, 20–24, 25–29, 30–34, 35–39, 40–44, 45–49 years), educational status (none, primary, secondary/technical, higher and non-formal), exposure to mass media (measured using access to radio and/or television), number of children ever born (0, 1–2, 3–4, 5 or more), currently working (no vs yes). The household characteristics included, the sex of the household head (male vs female), the religion of the household head (Christianity, Islam and others), ethnicity of household head (Hausa, Igbo, Yoruba and other ethnic groups) and the household wealth quintiles (poorest, poorer, middle, richer and richest). Community-level characteristics were explored using two variables, place of residence (urban vs rural) and community socio-economic status. Community refers to people living in the same locality (EAs). Community socio-economic deprivation was computed using a principal component analysis (PCA) comprising of wealth status (asset index in the poorest and poorer quintiles), unemployed, no formal education and illiteracy - those who cannot read at all. The standardised score was then categorised into tertiles (1 – least deprived communities to 3 – most deprived communities). Similarly, for the state-level factors, we extracted state socio-economic deprivation status using PCA and grouped into tertiles (1 – least deprived states to 3 – most deprived states) and also included regions (North Central, North East, North West, South East, South-South and South-West) (see supplementary Table 1).

### Data analysis

The descriptive analysis was presented using frequencies and percentages as well as the prevalence and 95% confidence interval (CI) of adequate and inadequate salt consumption. The prevalence was computed as the proportion of breastfeeding or pregnant women with adequate and inadequate iodine content in salt by women’s background characteristics. Similarly, the test of homogeneity of the proportion of inadequate iodised salt intake across the categories of each individual/household-, community and state characteristics were reported based on the corrected Pearson χ2 statistic. To account for the complex survey design, the Pearson χ2 statistic is transformed into an F-statistic with non-integer degrees of freedom using a second-order Rao and Scott correction [25, 26]. Then, five multi-level mixed effect log-binomial logistic regression models were fitted to the data in order of complexity. First, a null model (Model 1, with no explanatory variables) was fitted to the data to explore the variation due to community and state effects only. The second model (Model 2) included Individual and household variables, while the third model (Model 3) had only community characteristics. In the fourth model (Model 4), we examined only the state-level factors. The full model (Model 5) considered all Individual, community and state-level factors. The Loglikelihood, Akaike Information Criterion (AIC) and Bayesian Information Criterion (BIC) were used to assess the goodness of fit of the models. A lower value of the model statistic is said to be a better fit. More succinctly, if the difference between two information criteria (IC) value is greater than 10, this implies that the model with a smaller IC is superior, while a difference of 4 to 10 suggest a moderate superiority and a difference less than 4 implies that the two models are indistinguishable [27, 28]. All analyses were adjusted for the complex survey design and analysed using Stata 15.0 (StataCorp LLC, College Station, Texas, USA). Statistical Inferences were based on a 5% level of significance.

### Ethical consideration

This study was based on an analysis of a publicly available secondary dataset -the Multiple Indicator Cluster Survey; thus, no additional ethical clearance is required. Ethics approval was not required for this study since the data is secondary and is available in the public domain [21].

## Results

### Descriptive statistics

Data from a total of 4,911 pregnant and breastfeeding women were eligible for the study. One in ten women was an adolescent, and 56.7% had no or non-formal education (Table 1). Almost one in ten (10.8%) reported never having any childbirth, only 20.7% of the women were currently working, and 46.6% had no access to television or radio. About half of the household heads had no or non-formal education, were males (92.8%), were majorly Muslim (71.9%), and were from the Hausa ethnic group (60%). Wealth was evenly distributed among each category of the quintiles in the study population. For the community characteristics, 76.4% of the respondents resided in rural areas, and one-third were least deprived. The majority (seven in ten) of the respondents were from the Northern states, particularly, North-western region (44.4%), whereas 34.8% of respondents live in the least deprived state compared to 31.3% of the respondents who are living in the poorest states.

**Table 1:** Descriptive summaries of respondents’ background characteristics.

Variables	Frequency (%)
*Individual and Household characteristics*	
**Age group**	
15–19	502 (10.2%)
20–24	1,053 (21.4%)
25–29	1,273 (25.94%)
30–34	967 (19.7%)
35–39	653 (13.3%)
40–44	312 (6.3%)
45–49	151 (3.1%)
**Level of Education**	
None	1,579 (32.2%)
Primary	684 (13.9%)
Secondary/technical	1,140 (23.2%)
Higher	307 (6.3%)
Non-formal	1,200 (24.4%)
**Exposure to mass media**	
No	2,287 (46.6%)
Yes (TV/radio)	2,624 (53.4%)
**Children ever born**	
None	531 (10.8%)
1–2	1,621 (33.0%)
3–4	1,271 (25.9%)
5 or more	1,489 (30.3%)
**Currently working**	
Yes	1,016 (20.7%)
No	3,895 (79.3%)
**Sex of household head**	
Male	4,556 (92.8%)
Female	355 (7.2%)
**Religion of household head**	
Christianity	1,341 (27.3%)
Islam	3,530 (71.9%)
Others	40 (0.8%)
**Ethnicity of household head**	
Hausa	2,967 (60.4%)
Igbo	275 (5.6%)
Yoruba	455 (9.3%)
Other ethnic groups	1,214 (24.7%)
**Education of household head**	
None	1,217 (24.8%)
Primary	769 (15.7%)
Secondary/technical	1,007 (20.5%)
Higher	605 (12.3%)
Non-formal	1,314 (26.8%)
**Wealth status**	
Poorest	968 (19.7%)
Poorer	975 (19.8%)
Middle	984 (20.0%)
Richer	993 (20.2%)
Richest	992 (20.2%)
*Community characteristics*	
**Place of residence**	
Urban	1,159 (23.6%)
Rural	3,752 (76.4%)
**Socio-economic status**	
1 (Least deprived)	1,651 (33.6%)
2 (More deprived)	1,627 (33.1%)
3 (Most deprived)	1,633 (33.3%)
*State-level factors*	
**Socio-economic status**	
1 (Least deprived)	1,707 (34.8%)
2 (More deprived)	1,667 (33.9%)
3 (Most deprived)	1,536 (31.3%)
**Region**	
North Central	840 (17.1%)
North East	932 (19.0%)
North West	2,179 (44.4%)
South East	175 (5.6%)
South South	337 (6.9%)
South West	448 (9.1%)
**Total**	**4,911 (100%)**

### Prevalence of adequate and inadequate iodine content in salt

The prevalence of inadequate iodised salt consumption among pregnant and breastfeeding mothers was 35.2% (95% CI: 33.1–37.5), as shown in Table 2. Inadequate consumption of iodised salt was highest among pregnant and breastfeeding women aged 45–49 years (48.2%; 95%CI: 37.8–58.8), as well as those with non-formal education (52.7%; 95%CI: 47.7–57.6) and no education (34.6%; 95%CI: 31.3–38.1). Respondents who were exposed to mass media (30.0%), currently working (22.9%), and female headed-household (26.8%) had a lower prevalence of consuming salt with inadequate iodine compared to those with no access to mass media (41.2%), not working (38.4%) and male-headed household (35.9%), respectively. Wealth status was associated with intake of salt deficient in iodine; for example, 46.8% (95% CI: 42.1–51.5) of those in the poorest wealth quintile consume salt with inadequate iodine, while only 21.5% (95% CI: 17.6–25.9) among those in the richest quintile. Similarly, iodised salt intake was associated with community socio-economic deprivation and state socio-economic deprivation. For example, respondents in communities that are least deprived (23.0% vs 41.9%) and those in the least deprived states (22.6% vs 46.0%) had a lower prevalence of inadequate iodised salt intake compared to the respondent in the most deprived communities and states. Also, inadequate iodised salt intake was higher among respondents who reside in the rural (37.0% vs 29.3%) compared to urban areas and among those who are Hausas (p = 43.1%; 95%CI: 39.9–46.3) compared to all other ethnic groups. Almost one in ten pregnant or breastfeeding women in the North West consume salt with inadequate iodine. The corrected Pearson χ2 statistic also suggests that the proportion of inadequate iodised salt consumption differs across the categories of each background characteristics considered (p < 0.05 for all variables).

**Fig. 1:**
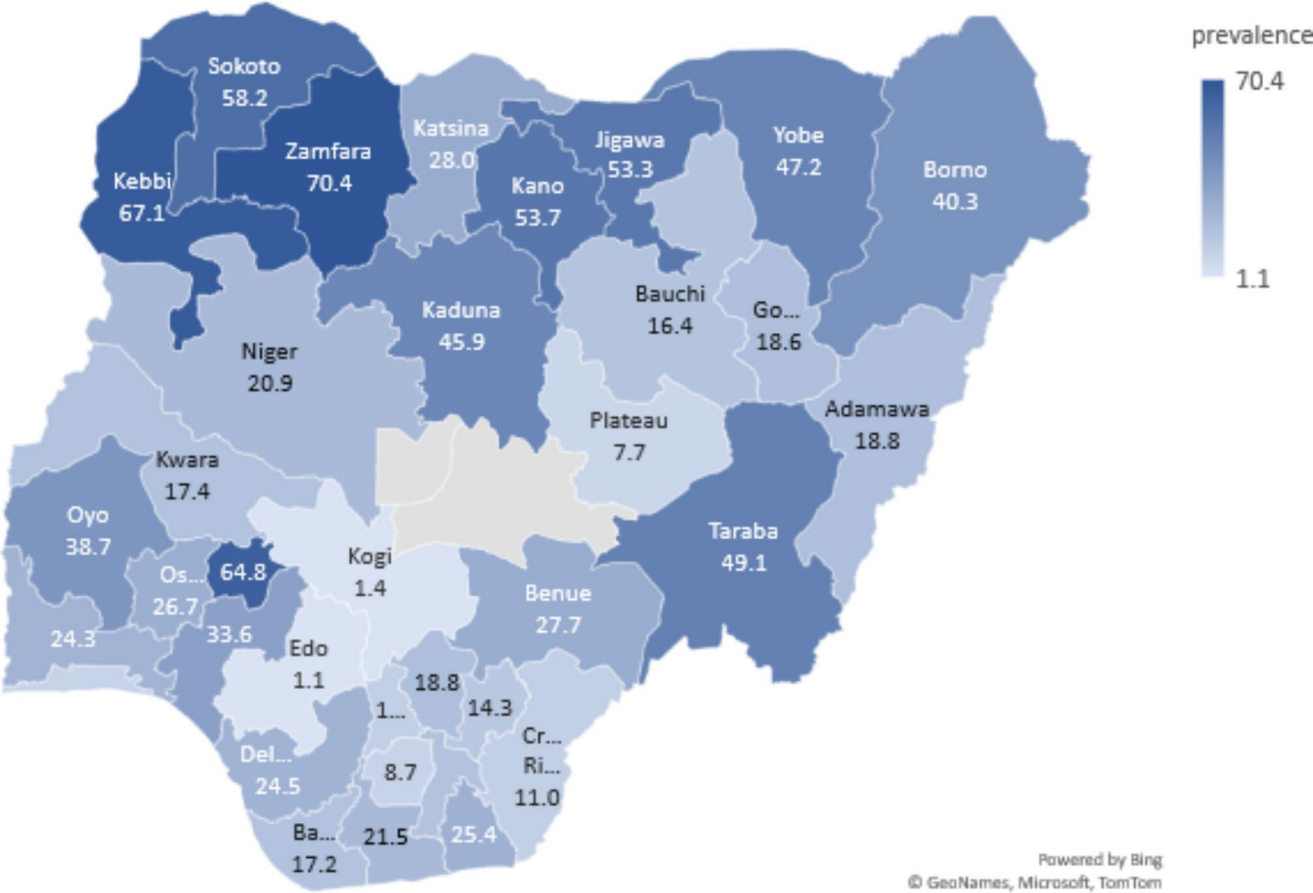
Geographical distribution of inadequate salt iodisation consumption among pregnant and breastfeeding mothers

Furthermore, the geographic distribution of inadequate iodised salt consumption among pregnant and breastfeeding mothers by states is shown in Fig. 1. The spatial map revealed the high burden of inadequate iodised salt intake in the Northwestern region; particularly with the highest burden in Zamfara (70.4%), Kebbi (67.1%) and Ekiti state (64.8%) in the Southwestern region.

**Table 2:** Prevalence of Iodised salt intake by women’s individual/household, community and state-level characteristics

*Variables*	*Prevalence (95% CI)*	*Prevalence (95% CI)*	*F-statistic* ^*†*^ *p-value*
	Adequate Iodized Salt	Inadequate Iodized Salt	
*Overall*	64.8(62.5–67.0)	35.2(33.1–37.5)	
*Individual and Household characteristics*			
**Age group**			p < 0.001
15–19	54.4(48.3–60.5)	45.6(39.5–51.7)	
20–24	68.1(63.7–72.1)	31.9(27.9–36.3)	
25–29	66.7(63.3–70.0)	33.3(30.0-36.7)	
30–34	68.3(64.4–72.1)	31.7(27.9–35.6)	
35–39	63.5(58.9–68.0)	36.5(32.0-41.2)	
40–44	60.1(52.7–67.0)	39.9(33.0-47.3)	
45–49	51.8(41.2–62.2)	48.2(37.8–58.8)	
**Level of Education**			p < 0.001
No education	65.4(61.9–68.7)	34.6(31.3–38.1)	
Primary	68.3(63.3–72.9)	31.7(27.1–36.7)	
Secondary/technical	75.3(71.6–78.8)	24.7(21.2–28.4)	
Higher	82.8(75.5–88.2)	17.2(11.8–24.5)	
Non-formal	47.3(42.4–52.3)	52.7(47.7–57.6)	
**Exposure to mass media**			p < 0.001
No	58.8(55.6–62.0)	41.2(38.1–44.4)	
Yes (TV/radio)	70.0(67.1–72.6)	30.0(27.4–32.9)	
**Parity**			p = 0.010
0 (None)	68.5(62.9–73.5)	31.5(26.5–37.1)	
1–2	67.1(63.9–70.2)	32.9(29.8–36.1)	
3–4	65.3(61.4–69.0)	34.7(31.0-38.7)	
5 or more	60.5(57.0-63.9)	39.5(36.1–43.0)	
**Currently working**			p < 0.001
Yes	77.1(73.7–80.1)	22.9(19.9–26.3)	
No	61.6(60.0-64.1)	38.4(35.9–41.0)	
**Sex of household head**			p = 0.005
Male	64.1(61.8–66.4)	35.9(33.6–38.2)	
Female	73.3(67.2–78.6)	26.8(21.4–32.8)	
**Religion of household head**			p < 0.001
Christianity	76.8(73.7–79.5)	23.2(20.5–26.3)	
Islam	60.1(57.2–62.9)	39.9(37.1–42.8)	
Others	76.1(61.6–86.3)	24.0(13.7–38.4)	
**Ethnicity of household head**			p < 0.001
Hausa	56.9(53.7–60.1)	43.1(39.9–46.3)	
Igbo	85.4(80.5–89.2)	14.6(10.8–19.5)	
Yoruba	74.4(69.0-79.2)	25.6(20.8–31.0)	
Other ethnic groups	75.7(71.8–79.2)	24.3(20.8–28.2)	
**Education of household head**			p < 0.001
None	71.3(67.7–74.7)	28.7(25.3–32.3)	
Primary	67.5(62.1–72.5)	32.5(27.5–37.9)	
Secondary/technical	70.7(66.7–74.4)	29.3(25.6–33.3)	
Higher	71.8(65.4–77.5)	28.2(22.5–34.6)	
Non-formal	49.3(44.6–54.2)	50.7(45.9–55.5))	
**Wealth status**			p < 0.001
Poorest	53.2(48.5–57.9)	46.8(42.1–51.5)	
Poorer	58.9(54.2–63.5)	41.1(36.5–45.8)	
Middle	64.1(58.6–69.2)	36.0(30.8–41.4)	
Richer	68.7(63.9–73.2)	31.3(26.8–36.1)	
Richest	78.5(74.1–82.4)	21.5(17.6–25.9)	
*Community characteristics*			
**Place of residence**			p = 0.003
Urban	70.7(66.4–74.6)	29.3(25.4–33.6)	
Rural	63.0(60.3–65.5)	37.0(34.5–39.7)	
**Socio-economic status**			p < 0.001
1 (Least deprived)	77.0(73.5–80.2)	23.0(19.8–26.5)	
2 (More deprived)	59.1(54.8–63.2)	40.9(36.8–45.2)	
3 (Most deprived)	58.1(54.0-62.1)	41.9(37.9–46.0)	
*State-level factors*			
**Socio-economic status**			p < 0.001
1 (Least deprived)	77.4(74.4–80.1)	22.6(19.9–25.6)	
2 (More deprived)	61.8(57.7–65.8)	38.2(34.2–42.3)	
3 (Most deprived)	54.0(49.6–58.3)	46.0(41.8–50.4)	
**Region**			p < 0.001
North Central	82.8(78.6–86.2)	17.3(13.8–21.4)	
North East	69.8(64.0–75.0)	30.2(25.0–36.0)	
North West	49.6(46.3–53.0)	50.4(47.0-53.8)	
South East	86.5(81.4–90.4)	13.5(9.6–18.6)	
South South	82.6(77.6–86.7)	17.4(13.3–22.4)	
South West	72.4(66.9–77.3)	27.6(22.7–33.1)	

^*†*^ *Corrected Pearson χ2 statistic accounting for the complex survey design*

### Multi-level Mixed effect log-binomial regression model

We fitted five multi-level mixed effect log-binomial regression models to our data. The null model (Model 1) showed a high intracluster correlation (ICC) of 45.3% (95%CI 40.3–50.3), an indication of greater dependency between levels. Similar results were obtained for models 2–5, suggesting the appropriateness of a multi-level approach. After adjusting for Individual and household characteristics in Model 2, we found a direct negative significant association between increasing wealth status and inadequate iodised salt intake. Respondents in the poorer, middle, richer and richest quintiles were 32%, 47%, 35% and 62% less likely to consume salt with inadequate iodine compared to those in the poorest households. Also, pregnant and breastfeeding mothers who were Igbos (aRR 0.29; 95%CI 0.16–0.54) and other ethnic groups (aRR 0.49; 95%CI 0.35–0.70) were less likely to consume salt with inadequate iodine compared to Hausas. However, respondents with no formal education were 1.8 times (95%CI: 1.36–2.42) more likely to consume salt with deficient iodine compared to those with no education, and households whose head had a secondary, higher, and non-formal education were 50%, 59% and 94% more likely to consume salt with inadequate iodine compared to those with no education.

In Model 3, we adjusted for the community socio-economic status. Pregnant and breastfeeding mothers residing in moderately and most deprived communities were 3.5 (95%CI: 2.57–4.73) and 4.7 times (95%CI: 3.38–6.55) more likely to consume salt with inadequate iodine than those from least deprived communities. Similarly, Model 4 showed that those residing in moderately and most deprived states were 1.9 (95%CI: 1.14-3.00) and 2.6 (95%CI: 1.50–4.44) times more likely to consume salt with inadequate iodine than those from least deprived states. Also, respondents from the North West and those from South West were 5.1 (95%CI: 3.18–8.30) and 2.9 (95%CI: 1.77–4.59) times more likely to consume salt with inadequate iodine compared to those from the North-Central region.

The fully adjusted model (Model 5), includes individual/household, community and state-level factors.

Our analysis showed that respondents with no-formal education were 1.7 (95%CI: 1.25–2.31) times more likely to consume inadequately iodised salt compared to those with no education. Similarly, household heads with no formal education (aRR 1.71; 95%CI: 1.16–2.52) and secondary education (aRR 1.44; 95%CI: 1.02–2.04) were associated with inadequate iodised salt intake compared to those with no education. Respondents in the middle (aRR 0.64; 95%CI: 0.42–0.97) and richest (aRR 0.51; 95%CI: 0.26–0.99) wealth quintiles were less likely to consume inadequately iodised salt compared to those in the poorest quintile. Also, respondents in the most deprived communities were 96% more likely to consume salt with inadequate iodine than those in the least deprived communities. Women in the Northwestern region and those from the Southwestern region were 4.0 and 3.5 times, respectively, more likely to consume salt with inadequate iodine compared to pregnant and breastfeeding women residing in the North-Central region. Results from the model fit revealed that Model 5 has the best fit based on loglikelihood (-2721.61) and AIC (5525.22) while the BIC showed that both Model 1 and Model 5 are indistinguishable (BIC: 5792.44 vs 5791.69) (Table 3).

**Table 3:** Factors associated with inadequate iodised salt intake among pregnant and breastfeeding mothers from multi-level logistic regression

*Variables*	*Null Model 1* ^*a*^	*Model 2* ^*b*^	*Model 3* ^*c*^	*Model 4* ^*d*^	*Model 5* ^*e*^
	*RR(95% CI)*	*aRR(95% CI)*	*aRR(95% CI)*	*aRR(95% CI)*	*aRR(95% CI)*
*Individual and Household characteristics*					
**Age group**					
15–19		Reference			Reference
20–24		0.84(0.56–1.28)			0.91(0.60–1.38)
25–29		0.92(0.61–1.39)			1.01(0.67–1.53)
30–34		0.77(0.49–1.22)			0.87(0.54–1.38)
35–39		0.89(0.55–1.44)			0.98(0.60–1.60)
40–44		1.24(0.71–2.15)			1.38(0.78–2.44)
45–49		1.37(0.71–2.63)			1.46(0.76–2.80)
**Level of Education**					
None		Reference			Reference
Primary		1.01(0.73–1.65)			1.30(0.84–2.03)
Secondary/technical		1.02(0.65–1.60)			1.26(0.72–2.20)
Higher		0.61(0.32–1.14)			0.78(0.38–1.59)
Non-formal		**1.81(1.36–2.42)**			**1.70(1.25–2.31)**
**Exposure to mass media**					
No		Reference			Reference
Yes (TV/radio)		0.86(0.68–1.07)			0.87(0.69–1.09)
**Parity**					
0 (None)		Reference			Reference
1–2		0.97(0.68–1.37)			0.94(0.66–1.09)
3–4		1.03(0.69–1.55)			0.96(0.64–1.45)
5 or more		1.01(0.65–1.56)			0.90(0.57–1.40)
**Currently working**					
No		Reference			Reference
Yes		0.83(0.63–1.09)			0.99(0.74–1.34)
**Sex of household head**					
Male		Reference			Reference
female		1.27(0.86–1.89)			1.30(0.88–1.94)
**Religion of household head**					
Christianity		Reference			Reference
Islam		1.07(0.76–1.52)			0.87(0.60–1.25)
Others		0.71(0.31–1.65)			0.70(0.29–1.68)
**Ethnicity of household head**					
Hausa		Reference			Reference
Igbo		**0.29(0.16–0.54)**			0.42(0.18–1.01)
Yoruba		0.93(0.58–1.50)			0.99(0.51–1.92)
Other ethnic group		**0.49(0.35–0.70)**			0.86(0.58–1.28)
**Education of Household head**					
None		Reference			Reference
Primary		1.30(0.90–1.89)			1.24(0.85–1.82)
Secondary/technical		**1.50(1.07–2.11)**			**1.44(1.02–2.04)**
Higher		**1.59(1.01–2.50)**			1.45(0.91–2.32)
Non-formal		**1.94(1.33–2.83)**			**1.71(1.16–2.52)**
**Wealth status**					
Poorest		Reference			Reference
Poorer		**0.68(0.48–0.97)**			0.75(0.53–1.06)
Middle		**0.53(0.36–0.80)**			**0.64(0.42–0.97)**
Richer		**0.65(0.43–0.98)**			0.86(0.54–1.38)
Richest		**0.38(0.22–0.67)**			**0.51(0.26–0.99)**
*Community characteristics*					
**Place of residence**					
Urban			Reference		Reference
Rural			0.79(0.57–1.08)		0.85(0.58–1.23)
**Socio-economic status**					
1 (Least deprived)			Reference		Reference
2 (More deprived)			**3.49(2.57–4.73)**		1.56(0.95–2.54)
3 (Most deprived)			**4.71(3.38–6.55)**		**1.96(1.04–3.72)**
*State characteristics*					
**Socio-economic status**					
1 (Least deprived)				Reference	Reference
2 (More deprived)				**1.85(1.14-3.00)**	1.27(0.75–2.17)
3 (Most deprived)				**2.58(1.50–4.44)**	1.65(0.90–3.01)
**Region**					
North Central				Reference	Reference
North East				1.29(0.71–2.35)	1.41(0.75–2.65)
North West				**5.14(3.18–8.30)**	**4.01(2.27–7.08)**
South East				0.88(0.50–1.56)	2.02(0.77–5.33)
South South				1.23(0.74–2.03)	1.57(0.89–2.76)
South West				**2.85(1.77–4.59)**	**3.46(1.79–6.68)**
Random effect					
ICC	45.3(40.3–50.3)	38.7(33.4–44.3)	43.2(38.1–48.4)	37.9(32.8–43.4)	37.5(32.2–43.1)
Model fit statistic					
Loglikelihood	-2906.564	-2764.482	-2851.675	-2778.321	-2721.608
AIC	5817.127	5590.964	5713.350	5574.642	5525.216
BIC	5830.126	5792.441	5745.846	5633.135	5791.685

*RR relative risk, aRR adjusted relative risk, CI confidence interval, ICC intracluster correlation*

*The aRR in bold implies significance at 5%*

^*a*^ *Null ModeI 1 – baseline model without any explanatory variables (unconditional model)*

^*b*^ *Model 2 – adjusted for only Individual and household-level factors*

^*c*^ *Model 3 – adjusted for only community-level factors*

^*d*^ *Model 4 – adjusted for only state-level factors*

^*e*^ *Model 5 – adjusted for individual and household-, community-, and state-level factors (full model)*

## Discussion

This study was expedient in light of the limited empirical literature on iodised salt consumption in Nigeria. The overall prevalence of inadequate iodised salt consumption among pregnant and lactating women in this study was 35.2%. The multi-level mixed-effect logistic model showed that pregnant and lactating women with non-formal education were more likely to consume inadequately iodised salt compared to those with formal education after adjusting for other multivariable factors. We found households with uneducated heads to be associated with increased risks of inadequate iodised salt consumption. This is likely due to nutritional education which is sometimes received by those with formal education as part of their usual lessons. In addition to formal education, health promotion interventions and non-formal education could be promising platforms for routing nutrition and its related educational and advocacy interventions. The importance of education to the consumption of iodised salt has been reported in studies from other sub-Saharan African countries such as Ethiopia [29] and Ghana [30]. Non-formal education in Nigeria targets children, youths and adults who either have dropped out of school or have never been to school [31].

Our findings are similar to those reported by Udofia, Yawson, Aduful et al [32] that women living in the most deprived communities were more likely to consume salt with inadequate iodine compared to those who reside in the least deprived communities. Without a strong negotiation and purchasing power, one can easily be persuaded to improvise with whatever they come across, even if they are fully aware of the adverse effects associated with that action [33]. A household survey in Ogun State revealed that most respondents were earning N100,000 ($232.3) per month with a mean income of N28,000 ($64.76). More than half of these households were spending N100($0.23) or less on iodised salt monthly [34].

Pregnant and breastfeeding women who belong to the top household wealth quintiles were less likely to consume inadequate iodised salt compared to those in the poorest category. Wealth or richness is associated with good nutrition and good health status, as empirical research suggests [35]. Wealthy persons usually tend to live in clean, hygienic and well-planned settlements, access quality healthcare and have frequent check-ups [36]. The indigent, however, is usually concerned with how to put food on the table and cater for the necessities of life as purported by Maslow’s Hierarchy of Needs [37]. These substantial variations may account for the findings in this study. Enhancing women’s economic opportunities by training them in various skills can increase their employability prospects and thereby make them economically sound to enable them to take the right nutrition, including iodised salt.

This study also revealed that those in Northwestern and Southwestern regions were more likely to consume salt with inadequate iodine compared to pregnant and breastfeeding women in the North- Central region. This suggests inequality in the distribution of iodised salt within Nigeria or disparity in the consumption pattern of iodised salt. As such, a thorough scrutiny of the distribution pattern will be worthwhile. Context appropriate education on iodised salt utilisation would be recommended in those regions with relatively low consumption of iodised salt. These educational campaigns can be channeled through widely accessed media channels such as radio or television. A survey in 2015 indicated that 99% of young people have social media accounts whilst 95.2% use smartphones to access various social media platforms. This may be suggestive that the social media can be used to target the young people for iodised salt consumption advocacies [38].

### Strengths and limitations of the study

Unlike previous studies on salt iodisation [24, 39, 40] this study focused on two key populations, pregnant and lactating women. It also followed rigorous and appropriate analytical procedures, thereby generating robust and reliable findings. The findings are also generalisable to all pregnant and lactating women in Nigeria, and its lessons/recommendations are useful for other sub-Saharan African countries. One of the major limitations of this paper is the cross-sectional nature of the study design, which do not allow for causal inference of the associated factors. WHO recommendation is not to have too much iodine in salt but a concentration of between 15 to 40 ppm of iodine. However, the classification of iodine content in salt samples in the MICS dataset into three main categories – 0 ppm, < 15 ppm and ≥ 15ppm limit our investigation to examine the range of iodine content in salt at the household level. More so, no individual woman was tested for iodine concentration, hence urinary iodine concentration test was not conducted. This would have provided additional insight into the iodine status of pregnant and breastfeeding mothers.

## Conclusion

The study revealed the prevalence of pregnant and lactating women in Nigeria with inadequate iodised salt consumption as well as other associated factors. Findings are suggestive that measures to overcome inadequate iodised salt consumption include ensuring equitable distribution of essential food commodities among the more and most deprived communities. Besides, there is the need to enhance women’s economic opportunities by training them in various skills that can increase their employability prospects and thereby making them economically sound to enable them to take proper nutrition, including iodised salt. Both formal and non-formal educational initiatives on nutrition are extremely important and should be prioritised by the Nigerian government in its efforts to encourage the consumption of iodised salt among pregnant and lactating mothers.

## Electronic Supplementary Material

Below is the link to the electronic supplementary material

Supplementary Material 1

## Data Availability

Data for this study were sourced from the Multiple Indicator Cluster Surveys and available here: https://mics.unicef.org/surveys

## Abbreviation

WHO: World Health Organization (WHO)
ICCIDD: International Council for the Control of Iodine Deficiency Disorders
MICS: Multiple Indicator Cluster Survey
AIC: Akaike Information Criterion
BIC: Bayesian Information Criterion
Ppm: Part per million
NISH2: National Integrated Survey of Households round 2
EA: Enumeration Areas
PSU: Primary Sampling Unit
PCA: Principal Component Analysis
CI: Confidence Interval
aRR: Adjusted relative risk
