# Supplementary material for: An assessment of Individual, community and state-level factors associated with inadequate iodised salt consumption among pregnant and lactating women in Nigeria
Source: BMC Pregnancy Childbirth. 2023 Jul 18;23:524. doi: 10.1186/s12884-023-05833-w (PMC10354909; doi:10.1186/s12884-023-05833-w)
Supplement: Supplementary file 1 — Supplementary Material 1 [file 12884_2023_5833_MOESM1_ESM.docx]

**Supplementary material**

**Supplementary Table 1.** Variable description, code and categorisation for analysis

| Variable | Variable code | Categorisation |
| --- | --- | --- |
| Inclusion variable |  |  |
| Pregnant | CP1 |  |
| Breastfeeding | UN11G |  |
| Outcome variable |  |  |
| Salt iodisation test | SI1 | Not iodised or <15 PPM = 0 and 15 PPM or more = 1 |
| Individual-level variables |  |  |
| Women age | WB2 | As coded in MICS dataset |
| Level of Education | WB4 | As coded in MICS dataset |
| Exposure to mass media | MT3 and MT4 | None (0), radio/tv (1) |
| Children ever born | CM10 | 0, 1-2, 3-4, ≥5 |
| Currently working | TA13B | As coded in MICS dataset |
| Household-level variables |  |  |
| Religion of head of HH | HC1A | As coded in MICS dataset |
| Sex of head of HH | HHSEX | As coded in MICS dataset |
| Ethnicity of head of HH | HC1C | As coded in MICS dataset |
| Education of head of HH | Helevel | As coded in MICS dataset |
| Wealth | Wealth | As coded in MICS dataset |
| Community level variable |  |  |
| Place of residence | HH6 | As in MICS dataset |
| Socio economic status  (wealth, education, literacy, occupation) | Wealth, WB4, WB7, TA13B | Operationalised through principal component analysis and grouped into tertiles |
| State/Regional level variable |  |  |
| Region | Zone |  |
| Socio economic status | Wealth, WB4, WB7, TA13B | Operationalised through principal component analysis and grouped into tertiles |
